# The importance of elderly people knowing basic first-aid measures

**DOI:** 10.1186/s12873-022-00675-9

**Published:** 2022-07-14

**Authors:** Eva Dolenc Šparovec, Damjan Slabe, Ivan Eržen, Uroš Kovačič

**Affiliations:** 1 Faculty of Health Sciences, Sanitary Engineering Department, Public Health Division, University of Ljubljana, Zdravstvena pot 5, 1000 Ljubljana, Slovenia; 2 National Institute of Public Health, Trubarjeva cesta 2, 1000 Ljubljana, Slovenia; 3 Faculty of Medicine, University of Ljubljana, Institute of Pathophysiology, Zaloška cesta 4, 1000 Ljubljana, Slovenia

**Keywords:** First aid, Elderly, Hypoglycaemia, Stroke, Out-of-hospital cardiac arrest

## Abstract

**Background:**

In the event of a sudden illness or injury, elderly individuals are often dependent on self-help and mutual assistance from partners. With poor access to medical services during natural and other disasters, the importance of first aid knowledge of elderly individuals increases even more. We assessed the opinions of different generations of Slovenian population regarding the importance of knowing the basic first aid measures. In addition, we aimed to examine the knowledge of first aid in the most common emergencies that threaten elderly people’s health and lives, focusing on the knowledge of elderly.

**Methods:**

A structured questionnaire was conducted with a representative Slovenian adult population (*n* = 1079). Statistically significant differences in average ratings of the importance of first aid knowledge were compared among different age groups with one-way ANOVA followed by a post hoc test. Significant differences in percentages of correct answers in particular cases of health conditions between different age groups were determined using the χ ^2 ^test followed by post hoc tests.

**Results:**

Slovenes are well aware of the importance of first aid knowledge and feel personally responsible for acquiring this knowledge. The general opinion is that older retirees need less first aid knowledge than individuals in younger populations. We found a high level of knowledge about symptoms and first aid measures for some of the most common health conditions that occur in old age. The level of knowledge in the group of the oldest respondents was comparable with that of younger age groups. However, their recognition of health conditions was also somewhat worse, especially when recognising the symptoms and signs of hypoglycaemia and heart attack. Most of the tested knowledge did not depend on a person’s age but on the time since that person was last educated in first aid.

**Conclusions:**

The knowledge of people older than 80 years is somewhat poorer than that in the younger population, mainly because too much time has passed since they were last educated in first aid. Public awareness of first aid needs to be increased and appropriate guidelines should be given with a focus on the elderly population.

**Supplementary Information:**

The online version contains supplementary material available at 10.1186/s12873-022-00675-9.

## Background

As the global population ages [1], the percentage of elderly patients accessing medical care increases [2]. Elderly people are a vulnerable group [1, 3] who face various health problems, such as hip injuries due to falls, hypoglycaemia in those with diabetes, stroke, and out-of-hospital cardiac arrest (OHCA) [1, 4]. Their vulnerability increases in disaster situations (natural or otherwise), exacerbating their prior chronic conditions [5–7]. In the case of OHCA and trauma, survival may rely on swift and correct first aid (FA) from bystanders [8–10]. Notably, most cardiac arrest victims were in their late sixties, and half of such cases are witnessed by a family member or friend who is usually over the age of 55 [11]. Therefore, knowledge of FA can be a great help to elderly people and promoting FA education among the elderly population appears imperative [4, 12]. We intended to assess the opinion of different generations of adults in the Slovenian population regarding the importance of knowledge of basic first aid measures among the elderly population.

A UK study showed that a relatively small proportion of elderly people had received FA education [13] and a Slovenian study reported that a more than 10 years had passed since they had participated in such training [14]. Notably, the first evidence-based guidelines for FA were developed only 22 years ago [15]. In addition, FA guidelines [16], especially basic resuscitation procedures [17], change frequently (every 5 years), so we can assume that the majority of elderly people have not been trained according to the currently recommended evidence-based FA procedures. An accurate assessment of the overall population’s baseline FA knowledge is required so that public FA education might be better tailored towards the elderly population. Therefore, we aimed to assess FA knowledge (with a particular emphasis on that in the elderly population) of the most common emergencies that might threaten the health of the elderly population in Slovenia. These data might help guide future public policies in improving FA knowledge in general and, potentially, the elderly population’s ability to perform FA.

## Methods

### Study design

A cross-sectional population-based survey was conducted in 2019 as part of a more extensive survey of Slovenian public opinion (SJM19/1), performed by the Public Opinion and Mass Communication Research Centre of the Faculty of Social Sciences of the University of Ljubljana. The survey population was a randomly selected sample of individuals aged 18 years and older from the overall Republic of Slovenia population (i.e., approximately 1,700,000 residents*)*. The selection of units (persons) included in the sample was carried out based on the Central Register of Population of Slovenia as a sample list. The sample selection was a random two-stage procedure, whereby each person in the population was equally likely to be included. In the first stage, 200 primary sampling units (PSUs) were selected using the Statistical Office of Slovenia’s Cluster of Enumeration Arias (CEA). The PSUs were randomly selected with a probability that was proportional to the CEA population size. PSU selection also considered previous stratification (of the different statistical regions and types of settlements). In the second stage, a fixed number (10 individuals including their names, surnames, year of birth, and addresses) was selected within each of the 200 PSUs (a total of 2000 individuals), using a simple random selection procedure. Thus, the size of the initial gross sample was 2,000 individuals (200 PSU * 10).

Of the 2000 individuals sampled, the response rate was 54% (*n* = 1,079; two respondents were partially surveyed). There were 139 inaccessible units in the sample (displaced, deceased, incorrect addresses, etc.), 403 refused to participate, and 379 refused for other reasons (rejection by another person, language barriers, health problems, etc.). All subjects were interviewed face-to-face by trained interviewers, and they completed a structured, pretested questionnaire. The interviewers interrupted the interviewees only to clarify a question if needed, but they did not reveal any information about the questions.

### Questionnaire

The structured FA questionnaire (see Additional file 1: Appendix A) consisted of demographic data as well as questions derived from preexisting empirical research conducted by the authors as well as a literature review. The first section of the questionnaire assessed the importance of FA knowledge and the responsibility for FA to specific groups in the population. The other two sections assessed general knowledge of the symptoms and signs of the most common health issues as well as the FA procedures for the latter. Two expert evaluators (methodologists with more than 10 years of experience in international research) pretested and evaluated the questionnaire.

### Statistical analysis

The data were analysed using the SPSS ver. 24 software package (SPSS Inc., Chicago, IL, USA); the demographic data were interpreted using descriptive statistics. For the elderly population, we selected persons older than 60 years, as the average retirement age in Slovenia was 60 years for women and 62 years for men between 2016 and 2020 [18] and according to the rigid definition of the elderly population with respect to an individual’s age [19]. The average ratings of assessing the importance of FA knowledge for specific groups and the importance of the responsibility for increasing FA knowledge for certain groups were assessed using a Likert scale (from 1-lowest grade to 5- highest grade) and expressed as the means ± SDs (standard deviations). For assessing the importance of FA knowledge for specific groups (see Table 2) we divided group of elderly population in young and old retirees. Young retirees are up to 80 years old. They are more actively involved in society. They join different organisations, go to various forms of physical activity, look after their grandchildren and have valid driving license. In the process of data collection, the interviewer explained such specifics. Statistically significant differences in average ratings among the different age groups were determined with one-way ANOVA followed by a post hoc Tukey test with appropriate adjustment of the *p* value for multiple comparisons. We performed preliminary analyses for normality. Given that the distribution was not normal, we also performed a nonparametric test (Kruskal–Wallis test). The results showed the same characteristics as the ANOVA results and did not change the values ​​of the results. Therefore, we presented the ANOVA results for a more straightforward interpretation and comprehensibility of the averages compared to the ranks. Dependent variables obtained with substantive questions are expressed as the percentages (%) of affirmative/correct answers, and then the statistically significant differences among the different categories of age groups (independent variable) were determined using the χ^2^ square test followed by appropriate post hoc tests for multiple comparisons. Due to multiple comparisons, the level of statistical significance was adjusted (*p* < 0.05). With regression analysis, we determined the correlation among the variables of FA knowledge (achieved number of points on the knowledge test), the time that had elapsed since the last FA training (on ranked values) and the age (in years) of the respondents. We performed preliminary analyses for normality. Given that the distribution was not normal, we used Spearman’s rho.

## Results

The demographic data of 1,079 respondents are presented in Table 1. Most respondents (37%) were from the age group older than 60 years. Just under a third of the respondents had a secondary or higher education. Almost half of those over 60 years of age (198/402) were under the age of 70, whereas 60 out of the 402 (15%) respondents in the group over 60 years of age were older than 80. Of the respondents over 60 years of age, 29% had a primary education or less, 26% had a secondary school education (vocational education), 22% had a secondary school (academic education), and 23% had a higher education. More than half of all respondents had undergone FA training 10 (or more) years ago, whereas this was the case for 72% of those over 60 years old. In addition, 11% of the elderly population had never had such training.

**Table 1:** Demographic data

SEX	all (*n* = 1079)
male	49% (*n* = 529)
female	51% (*n* = 550)
AGE (years)	all (*n* = 1079)
less than 30	16% (*n* = 169)
from 31 to 45	23% (*n* = 246)
from 46 to 60	24% (*n* = 262)
61 and more	37% (*n* = 402)
AGE (years) in group older than 60 years	*N* = 402
61 to 69	51% (*n* = 205)
70 to 79	34% (*n* = 137)
80 and more	15% (*n* = 60)
EDUCATION	all (*n* = 1079)
primary school	17% (*n* = 183)
secondary school – vocational education	22% (*n* = 237)
secondary school – academic education	31% (*n* = 335)
higher education—college, university	31% (*n* = 335)
NUMBER OF YEARS FROM LAST FA COURSE	all (*n* = 1079)
1 and less	13% (*n* = 140)
from 1 to 5	18% (*n* = 194)
from 6 to 10	9% (*n* = 97)
more than 10	55% (*n* = 594)
never	5% (*n* = 54)
NUMBER OF YEARS FROM LAST FA COURSE in group older than 60 years	*N* = 402
1 and less	5% (*n* = 20)
from 1 to 5	8% (*n* = 32)
from 6 to 10	3% (*n* = 12)
more than 10	73% (*n* = 294)
never	10% (*n* = 40)

The mean level of assessment of the importance of FA knowledge on the Likert scale (from 1-lowest grade to 5- highest grade) was 4.49 ± 0.607, indicating that adult Slovenians generally believed that FA knowledge was important or very important. In all age groups of the respondents, the average levels of the importance of FA knowledge attributed to students and employed people, respectively, were statistically significantly (*p* < 0.05) higher when compared to older retirees and children (Table 2). People over the age of 46, compared to those under the age of 30, gave a higher score for the importance of FA knowledge for children and adolescents. Other differences in the assessment of the importance of FA knowledge for different population groups among different age groups were not statistically significant (Table 2).

**Table 2:** Respondents’ assessments of the importance of FA knowledge for certain age groups of the population

POPULATION	^&^all	AGE GROUPS OF RESPONDENTS (years)				
	(*n* = 1079)	< 30 ys (*n* = 169)	31–45 ys (*n* = 246)	46–60 ys (*n* = 262)	> 60 ys (*n* = 402)	^#^*P*
Children and adolescents	4,29 ± 0,25	4,11 ± 0,95	4,24 ± 0,97	4,37 ± 0,87*	4,34 ± 0,94*	,020
Students and employed	4,77 ± 0,51^&^	4,84 ± 0,44	4,77 ± 0,5	4,73 ± 0,55	4,77 ± 0,51	,177
Younger retirees	4,64 ± 0,65	4,64 ± 0,58	4,59 ± 0,64	4,62 ± 0,636	4,67 ± 0,65	,531
Older retirees	4,27 ± 0,92	4,19 ± 0,88	4,28 ± 3,94	4,33 ± 0,86	4,26 ± 0,92	,469

Level of importance of FA knowledge was assessed on a Linkert scale from 1 to 5 (1 means not important at all and 5 means very important) and presented as average ± SD

^#^*p* value comparing different age groups of respondents (ANOVA); * statistically significantly different from the group up to 30 years after performing post hoc comparison

^&^the attribution of the importance of FA knowledge to students and employed people is statistically significantly (*p* < 0.05) higher than attribution to the importance of FA knowledge for older retirees and children and adolescents

In general, respondents attributed the responsibility of the selected stakeholders involved in the dissemination of FA knowledge (rated on a scale of 1 to 5) to all subjects as important or very important (mean = 4.2; standard deviation = 0.07). The respondents attributed a statistically significantly (*p* < 0.05) higher responsibility for the dissemination of FA knowledge to different organisations or individuals than to the media or personal physicians (Table 3). There was no statistically significant difference (*p* > 0.05) between the level of responsibility of organisations and individuals. The different age groups of respondents were almost unanimous in this opinion, as there were no significant differences among them (*p* > 0.05), except in the case of a personal physician, for which the ANOVA test showed a significant global effect, which was not confirmed by the post hoc tests (Table 3).

**Table 3:** Respondents’ assessments of the attributed responsibility of the selected stakeholders involved in the dissemination of FA knowledge

STAKEHOLDERS	^&^ ALL (*n* = 1079)	AGE GROUPS OF RESPONDENTS				
		< 30 ys (*n* = 169)	31–45 ys (*n* = 246)	46–60 ys (*n* = 262)	> 60 ys (*n* = 402)	^#^*p* value
Personal doctor	4,08 ± 1,14^&^	3,97 ± 1	3,98 ± 1,21	4,05 ± 1,15	4,20 ± 1,14	0,042 ^*^
Mass media	3,90 ± 0,99^&^	3,88 ± 0,98	3,87 ± 1,05	3,95 ± 0,96	3,90 ± 0,98	0,8
Organizations	4,35 ± 0,89	4,43 ± 0,77	4,37 ± 0,86	4,31 ± 0,85	4,34 ± 0,97	0,57
Individual	4,33 ± 0,93	4,42 ± 0,84	4,33 ± 0,88	4,29 ± 0,89	4,33 ± 1,03	0,58

Level of the attributed responsibility of the selected stakeholders involved in the dissemination of FA knowledge was assessed on a Linkert scale from 1 to 5 (1 means not important at all and 5 means very important) and presented as average ± SD

^#^*p* value comparing different age groups of respondents (ANOVA); *—no significant differences could be found after pairs of individual age groups of respondents were compared by post hoc test;

^&^Assessment levels of responsibility for FA knowledge to the media or a personal physician are statistically significantly (*p* < 0.05) lower than to organizations and individuals. There are no statistically significant differences between organizations or individuals (*p* > 0.05)

We tested the correlation among the variables of FA knowledge (combined from the number of points achieved on the knowledge test; maximum total score was 14), the time since the last FA training (time categories) and the age (years) of the respondents with regression analysis. The correlation between FA knowledge and the time elapsed since the last FA training was negative but negligible (correlation strength -0.083). Nevertheless, the correlation was statistically significant (*p* < 0.001). In contrast, the number of points scored on the knowledge test was not correlated with the age of the respondents (correlation strength 0,029; *p* = 0.346).

A very high number of respondents in all age groups recognised the signs and symptoms of a stroke and heart attack and were aware of the correct FA measures for these conditions (Table 4). In contrast, the proportion of correct answers was low in the case of hypoglycaemia and was by far the lowest (16%) in the case of providing FA to an individual who shows no signs of life. The respondents older than 60 years less frequently recognised the signs and symptoms of hypoglycaemia compared to those in the group aged 46 to 60 years (χ2 = 31.196; *p* < 0.001). The share of correct answers in the oldest age group was also statistically significantly lower than that in the group aged younger than 46 years (*p* < 0.001). Interestingly, although they less frequently recognised this disorder, those over 60 years of age were nevertheless more frequently aware of the correct FA measures for hypoglycaemia compared to those younger than 31 years (χ2 = 45.625; *p* < 0.001). (Table 4).

**Table 4:** Recognition of health conditions (emergencies) and knowledge of correct emergency FA measures in respondents from different age groups

	AGE GROUPS OF RESPONDENTS (years				
	< 30 ys (*n* = 169)	31–45 ys (*n* = 246)	46–60 ys (*n* = 262)	> 60 ys (*n* = 402)	ALL (*n* = 1079)
PROPORTION OF CORRECT ANSWERS ABOUT RECOGNITION OF HEALTH CONDITION					
Hypoglycaemia*	72%	68%	74%	55% ^&^	65%
Stroke	91%	89%	90%	87%	89%
Heart attack*	94%	94%	95%	89%	93%
PROPORTION OF CORRECT ANSWERS ABOUT FA MEASURES					
Hypoglycaemia*	43%	60%	68%	71%^#^	63%
Stroke	98%	98%	99%	97%	99%
Heart attack	90%	90%	87%	90%	89%
*CPR*	19%	18%	17%	13%	16%

*CPR* – *“Which measure is most important if we find ourselves with a person who shows no signs of life?”*

^*^ There is a statistically significant difference in the proportion of correct answers between age groups

^&^statistically significantly (*p* < 0.001) less than in other age groups of respondents

^#^statistically significantly (*p* < 0.001) different than group < 30 years old

Taking a closer look at the knowledge of people over 60 years of age (Table 5), we found that people aged 61 to 69 years had more knowledge than people over 80 years of age in the recognition of hypoglycaemia (χ2 = 7.018; *p* = 0.03). The same was true for recognising a heart attack (χ2 = 20.459; *p* < 0.001; Table 5). Most respondents would prefer to use an AED over chest compressions when providing FA to a person who shows no signs of life. The proportion of those older than 80 is greater than in younger age groups but was statistically insignificant (χ2 = 2.561; *p* = 0.278).

**Table 5:** Recognition of emergency medical conditions and knowledge of emergency FA measures among respondents over 60 years of age

	AGE GROUPS OF RESPONDENTS (years)			
	61–69 ys (49.3%, *n* = 198)	70–79 ys (34.8%, *n* = 140)	> 80 ys (15.9%, *n* = 64)	ALL (*n* = 402)
PROPORTION OF CORRECT ANSWERS ABOUT RECOGNITION OF HEALTH CONDITION				
Hypoglycaemia^a^	61%	53%	42%^#^	55%
Stroke	86%	91%	80%	87%
Heart attack^a^	91%	94%	73%^&^	89%
PROPORTION OF CORRECT ANSWERS ABOUT FA MEASURES				
Hypoglycaemia	76%	70%	61%	71%
Stroke	98%	96%	98%	97%
Heart attack	88%	89%	97%	90%
*CPR*	11%	14%	19%	13%

*CPR – “Which measure is most important if we find ourselves with a person who shows no signs of life?”*

^a^There is a statistically significant difference in the proportion of correct answers between age groups

^#^statistically significantly (*p* = 0.03) smaller proportion than in respondents 61–69 years old

^&^statistically significantly (*p* < 0.001) smaller proportion than in both groups of respondents younger than 80 years

## Discussion

A layperson, by providing FA, can have a significant impact on improving health and survival in a medical emergency [8–10]. However, an FA intervention can only be effective if provided by a layperson with the confidence, willingness, skills and knowledge to do so [20]. As such, our finding that all respondents rated FA knowledge as being very important for all age groups is encouraging and in line with previous research suggesting that, in present day Slovenia, providing FA is considered a high moral duty [21]. Most respondents assessed that they were personally responsible for obtaining awareness of FA measures, as well as traditional organisations such as the Red Cross, with less responsibility attributed to either the media or physicians. Nevertheless, a notable finding in this study was that most responders reported that FA knowledge was less important for older retirees compared to the rest of the adult population. Furthermore, older individuals are typically more vulnerable (psychologically/medically/socially), especially in emergencies, such as a pandemic [22–25].

Most adults in Slovenia correctly recognised the signs and symptoms of a stroke and heart attack and were aware of the correct FA measures in these situations. This finding is in accordance with similar surveys from Europe [26, 27] and New Zealand [28]. Our finding that respondents under the age of 30 possessed a high degree of FA knowledge contrasts with the results of a study conducted in 9 European countries reporting that younger individuals recognised fewer heart attack symptoms than individuals in older age groups [27]. Cardiac patients (who are common among the elderly population) had broader knowledge about stroke symptoms and risk factors than the general population [26], which was again consistent with our findings that elderly individuals are not inferior in recognising and responding to stroke and heart attack.

The findings regarding the recognition of a stroke and heart attack contrast with those regarding hypoglycaemia; the latter is less well recognised by elderly individuals in comparison to younger respondents, although they tend to apply the correct FA countermeasures. Interestingly, individuals younger than 30 recognised the symptoms of hypoglycaemia but were less well equipped in applying the correct FA measures. Other studies [29, 30] also suggested poor general knowledge about diabetes as well as of the causes and care of hypoglycaemia among the elderly population. In a related study, Turk et al. [31] concluded that diabetic people in Slovenia (who are most often elderly) reported a relatively low level of general knowledge about their disease.

When faced with a person who shows no signs of life, the overwhelming majority of respondents in Slovenia had difficulty determining the correct CPR procedure to follow. Most respondents incorrectly answered to use an AED instead of chest compressions [17]. Heard et al. [23] determined that while belief in one’s skills was high (70%), their actual knowledge was lower, particularly for CPR-related knowledge and skills, as evidenced by the fact that only 5% of trained individuals knew the correct compression-to-ventilation ratio. Another study [32] that compared bleeding control and CPR revealed that while most respondents had a good understanding of bleeding control (87%), few were familiar with CPR (21%). Similarly, in the present study, only 16% of the respondents were aware of the correct CPR measures when faced with a person showing no signs of life. Richman et al. [33] reported that those in a senior living community had poor understanding of the use of AEDs in CPR. Poor CPR knowledge among the elderly population was also confirmed by others [34, 35]. Of note OHCA patients in 2020 were typically older and suffered from chronic medical conditions such as hypertension, diabetes and physical limitations [36]. The use of medical emergency services was significantly reduced in March 2020 [37], as was as the proportion of OHCA patients who survived until their emergency admission during the pandemic period [23, 38]. Factors associated with a reduced OHCA survival rate were a prolonged time of arrival of emergency medical services, a higher proportion of OHCAs occurring at home and a low proportion of individuals nearby who were capable of providing adequate CPR.

If we examine the knowledge of people over the age of 60 years in more detail, the proportions of correct answers about the recognition of hypoglycaemia and heart attack in our survey were statistically significantly smaller among the oldest respondents (over 80 years old). Because the oldest people are the most vulnerable and fragile [24, 25] and are often left to fend for themselves, this can lead to adverse health outcomes. Although elderly individuals are aware of the importance of FA knowledge, their (too) low self-confidence about this knowledge is often a problem [13, 34]. A reason may be that they are less likely to be trained or show a willingness to be trained in FA measures. Regardless of the reason for their lower readiness to participate in FA courses, longer periods of time since the last participation in an FA course correlate with poor FA knowledge, which follows the results of our study. Notably, Caap and colleagues [39] linked the low level of CPR knowledge to the fact that the majority (57%) of the respondents had not yet attended a CPR course. Accordingly, in our survey, 55% and 73% of all respondents and those older than 60 years, respectively, attended their most recent FA course more than 10 years ago. Furthermore, 5% and 10% of them, respectively, had never attended an FA course.

### Limitations

One of the limitations of our research is that we only studied the theoretical knowledge of FA. Behaviour in real situations could be different. In real situations of cardiac arrest, Park et al. [40] found that people over 60 years of age performed lower quality CPR. Notably, Takei and colleagues [41] confirmed that, in reality, older eyewitnesses are less likely to perform CPR. The reasons and sources of acquiring FA knowledge in other medical situations could also be explored in more detail. The non-validated questionnaire is another limitation of our research. In addition, one might argue that we did not focus on a representative sample of the elderly population, although the total number of respondents older than 60 years was not small (*n* = 402). In contrast, we deliberately conducted a survey with the entire adult Slovenian population because it allowed us to compare different age groups.

## Conclusions

The elderly population is a vulnerable group dependent on self-help and mutual assistance from partners in the event of sudden illnesses or injuries. With poor access to emergency medical services during natural and other disasters (e.g., due to the COVID-19 pandemic), the importance of the elderly population’s FA knowledge increases even more. It is encouraging that we found solid knowledge among the elderly population regarding some emergencies in our research. The low level of knowledge regarding the understanding of procedures of CPR with AEDs and recognising hypoglycaemia poses a challenge and commitment to engaging key stakeholders in the training of laypeople in FA. In the future, significant emphasis should be placed on those over 80 years of age who are exposed the most to sudden illnesses and injuries.

## Supplementary Information

**Additional file 1: Appendix A. **Survey questionnaire: The first section of the structured FA questionnaire assessed the importance of FA knowledge and the responsibility for FA to specific groups in the population. The other two sections assessed general knowledge of the symptoms and signs of the most common health issues as well as the FA procedures for the latter.

## Data Availability

The datasets generated and analysed during the current study are available in the ADP – Social science data archives repository: https://doi.org/10.17898/ADP_SJM191_V1

## Abbreviations

FA: First aid
OHCA: Out-of-hospital cardiac arrest
CPR: Cardiopulmonary resuscitation
AED: Automated external defibrillator
SD: Standard deviation
