# Supplementary material for: The importance of elderly people knowing basic first-aid measures
Source: BMC Emerg Med. 2022 Jul 14;22:128. doi: 10.1186/s12873-022-00675-9 (PMC9281334; doi:10.1186/s12873-022-00675-9)
Supplement: Supplementary file 1 — Additional file 1: Appendix A. Survey questionnaire: The first section of the structured FA questionnaire assessed the importance of FA knowledge and the responsibility for FA to specific groups in the population. The other two sections assessed general knowledge of the symptoms and signs of the most common health issues as well as the FA procedures for the latter. [file 12873_2022_675_MOESM1_ESM.docx]

**Appendix A. SURVEY QUESTIONNAIRE**

**1. On a scale of 1 to 5 (where 1 means “not important at all” and 5 means “very important”) rate the importance of FA knowledge for certain age groups of population.**

| children and adolescents | 1 | 2 | 3 | 4 | 5 |
| --- | --- | --- | --- | --- | --- |
| students and staff | 1 | 2 | 3 | 4 | 5 |
| younger retirees | 1 | 2 | 3 | 4 | 5 |
| older retirees | 1 | 2 | 3 | 4 | 5 |

**2. On a scale from 1 to 5 (where 1 means “not important at all” and 5 means “very important”), assess how important each of the subjects of the selected stakeholders involved in the dissemination of first aid knowledge is for raising the knowledge of first aid measures in Slovenia in case of sudden illnesses and injuries.**

| personal doctor | 1 | 2 | 3 | 4 | 5 |
| --- | --- | --- | --- | --- | --- |
| mass media (TV, radio, internet) | 1 | 2 | 3 | 4 | 5 |
| different organizations (npr. Red Cross, AMZS..) | 1 | 2 | 3 | 4 | 5 |
| individual | 1 | 2 | 3 | 4 | 5 |

**3. Which of the following medical emergencies is most likely to occur in a person who is hungry, sluggish, sweaty, pale, shaky, and has a thinking disorder (difficulty concentrating, fatigue, confusion)?**

**4. Which of the following medical emergencies is most likely to occur in a person who speaks indistinctly, has a drooping corner of the mouth and a paralyzed half of the body?**

**5. Which of the following medical emergencies is most likely to occur in a person who has severe chest tightness and left arm pain, shortness of breath, nausea, and cold sweats?**

Possible answers to questions 3, 4, 5:

- stroke
- hypoglycaemia
- heart attack
- severe allergic reaction (anaphylaxis)

**6. What would you give to a person who is conscious and has complications of diabetes (hypoglycemia)?**

- chocolate
- water
- sugar (1 tablespoon)
- a cup of herbal tea
- nothing

**7. What would you do first if you recognized a conscious person with stroke in the evening time?**

**8. What would you do first if you recognized a conscious person with heart attack in the evening time?**

Possible answers to questions 7, 8:

- I would call 112 immediately.
- I would wait until morning and take the person to the doctor myself.
- I would start with cariopulmonary resusciation.
- I would give a person a cup of herbal tea.

**9. Which measure is most important if we find ourselves with a person who shows no signs of life?**

- chest massage with circular movements on the skin and compresses on the forehead
- stable position for the unconscious person.
- rescue breaths
- use of a device Automatic external defibrillator
- strong and deep compressions on the middle of the chest

**DEMOGRAPHIC DATA**
